# Cross-cultural adaptions and measurement properties of the WORC (Western Ontario rotator cuff index): a systematic review

**DOI:** 10.1186/s12955-020-1276-9

**Published:** 2020-01-29

**Authors:** Rochelle Furtado, Joy C. MacDermid, Goris Nazari, Dianne M. Bryant, Kenneth J. Faber, George S. Athwal

**Affiliations:** 1 Physiotherapy, Health and Rehabilitation Science, Western University, London, ON Canada; 2 Collaborative Program in Musculoskeletal Health Research, Bone and Joint Institute, Western University, London, ON Canada; 3 Roth McFarlane Hand and Upper Limb Centre, St. Joseph’s Hospital, London, ON Canada

**Keywords:** Rotator cuff disorders, Translation, Psychometric properties, WORC, Quality of life, Patient reported outcomes, Shoulder, Rotator cuff tear

## Abstract

**Background:**

To evaluate the translations, cross-cultural adaptation procedures and measurement properties of the Western Ontario Rotator Cuff Index (WORC), when it is adapted for different cultures.

**Methods:**

A systematic review was performed, considering different cultural adaptions of the WORC accessible through MEDLINE, CINAHL, EMBASE and/or Google Scholar. Included were prospective cohort studies that used an adapted version of the WORC to measure QoL in patients with rotator cuff disorders. All studies were evaluated according to the current guidelines for cross-cultural adaptations and measurement properties.

**Results:**

The search retrieved 14 studies that met the inclusion criteria. According to the recommended guidelines for cross-cultural adaptations, 8 studies performed 100% of the steps, 2 studies performed 80% of the steps and 4 studies used previously translated measures. When evaluating the studies’ psychometric properties based on the quality criteria, none of the studies reported all recommended measurement properties. All of the studies reported the measurement property of reliability, but none of the studies reported agreement. Internal consistency was fully reported by 15% of studies. Construct validity was reported by 43% of studies. Only one study reported 100% of the cross-cultural adaption guidelines and 83% of the quality criteria.

**Conclusions:**

Although the majority of studies demonstrated proper adaptation procedures, testing of the measurement properties were inadequate. It is recommended that the current adapted versions of the WORC undergo further testing before use in clinical practise, and researchers continue to adapt the WORC for different cultures as it proves to be an appropriate instrument for assessing rotator cuff pathology.

## Introduction

Shoulder pain is one of the most commonly reported musculoskeletal problems that result in the restriction of work and/or social activities [1–3]. Rotator cuff disorders (RCDs) are the most common causes of shoulder pain, as chronic tendon degeneration of the cuff results in a loss of tendon integrity that ranges from partial to massive tears [3]. RCDs are highly prevalent in males, and more frequent in working individuals over the age of 60 [2, 3]. Overall, untreated RCDs eventually lead to the loss of quality of life (QoL) [1–3].

Measuring QoL can help to determine prognosis and evaluate treatment outcomes in patients with RCDs [2–4]. In order to estimate QoL, self-reporting through patient reported outcomes (PROs) [1–4] is required. The Western Ontario Rotator Cuff Index (WORC), developed by Kirkley et al. is one of the most validated disease-specific questionnaires to measure QoL in patients with RCD [5]. The WORC focuses on 5 domains; 1) pain and physical symptoms (6 items), 2) sports and recreation (4 items), 3) work (4 items), 4) lifestyle (4 items), and 5) emotions (3 items). The WORC has a total of 21 items that respondents answer on a visual analogue scale, with anchors of “no pain/difficulty and extreme pain/difficulty”. Each item has a possible score from 0 to 100, and summated to a total score of 0–2100, with a higher score representing a poor QoL. Items chosen for the WORC were derived from a variety of published health status scales, discussions with healthcare professionals, and interviews with a variety of patients with rotator cuff pathology [4–7].

While there are a variety of PROs for evaluating and detecting changes in a patient’s clinical condition over time, most were developed in English [6–8]. Due to the increasing globalization and importance of using these tools across cultures, researchers have been directed towards the translation of these outcome measures [6, 7]. The availability of PROs for different cultures is not only economical but can facilitate future comparisons among different populations; as long as the translated equivalent is successful [8]. Therefore, PROs need to be accurately translated, cross–culturally adapted and assessed for their psychometric measurement properties [7, 8].

For an adapted measure to be applied to the intended population, careful attention to word change and question structure is required [6–8]. The cross-cultural adaption process, verifies the equivalence with the original version and resolves any cultural or health differences amongst countries [6, 9]. Additionally, it is also important to evaluate the psychometric properties of the adapted measure [9, 10]. Evaluation after translation can verify if the adapted measure retains the psychometric properties of the original, as discrepancies between cultures can influence the results [6, 8–10]. Therefore, guidelines have been developed to help researchers critically analyze these studies [6, 10–12].

Although the WORC has strong psychometric properties [1, 2, 13] in an English context, there is a concern regarding the cross-cultural adaptation procedures and measurement properties when translated. As prior research has shown, it is critical to evaluate PROs before their use in a clinical setting. Therefore, this systematic review aims to evaluate the translations, cross-cultural adaptation procedures and measurement properties of the WORC, when adapted for different cultures.

## Methods

### Study selection

**Fig. 1:**
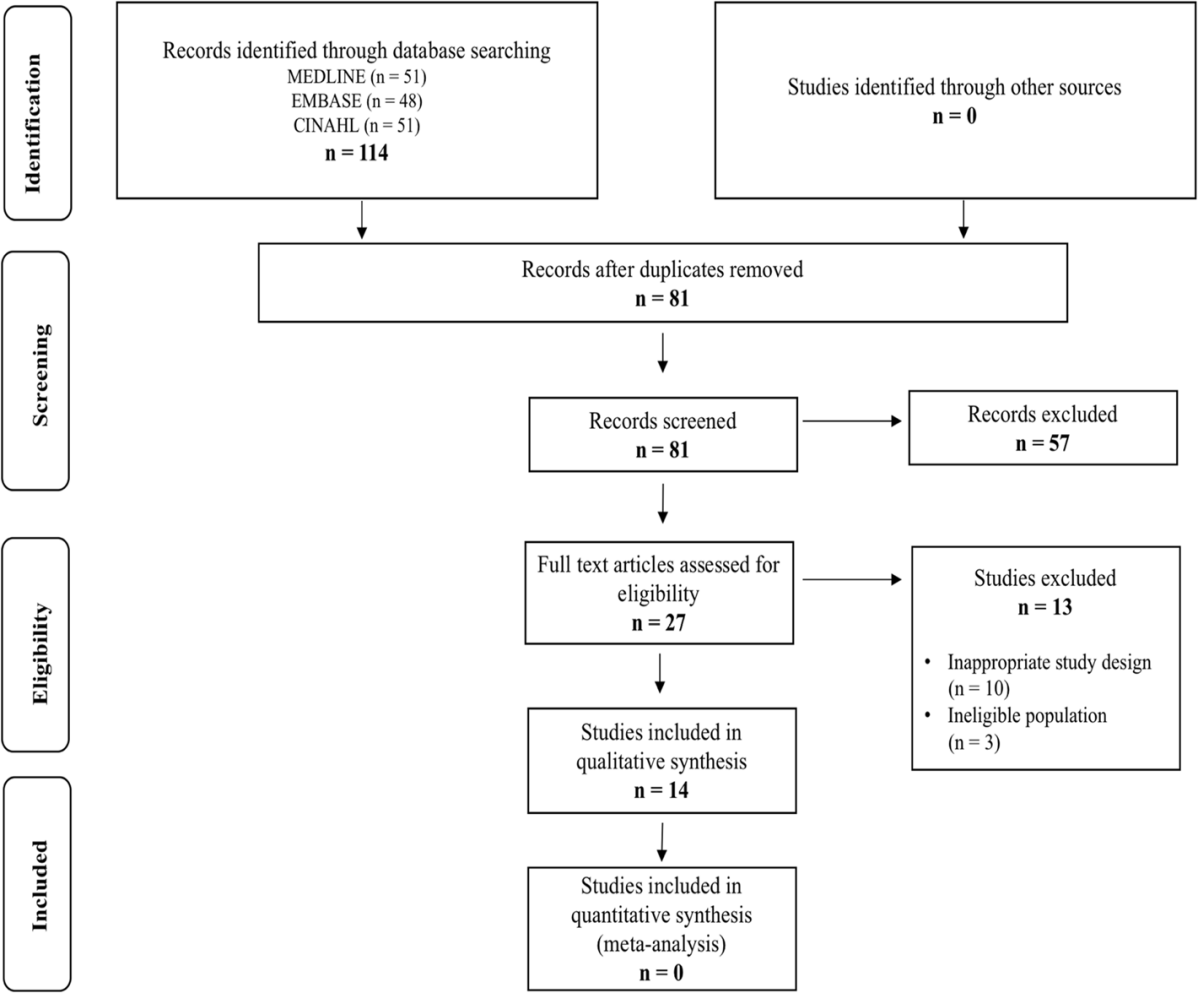
Flow diagram of literature search

We conducted a systematic review of studies that addressed the translation process and psychometric testing of the WORC in different cultures. The systematic searches were performed in the following key electronic databases: MEDLINE (Ovid), EMBASE, EBSCO- Host (CINAHL), and Google Scholar. Search terms and Boolean operators (AND or OR) used were: Western Ontario Rotator Cuff Index AND validation OR translation OR cross-cultural adaption AND different languages (e.g., German). This search strategy and electronic databases are frequently reported in other systematic reviews. The searches were not limited by publication date. The final search was April 12th, 2019, and registered on PROSPERO. (No.CRD42018100201) A flow diagram of the search strategies are provided in Fig. 1, according to Moher et al. [14].

### Inclusion criteria

Studies were considered eligible for inclusion if they assessed a cross-cultural adaption of the WORC and its measurement properties in a specific language. Studies must be published as a full manuscript in a peer – reviewed journal. Thesis and dissertations, books and abstracts from conferences were excluded. There were no language restrictions.

### Data extraction and analysis

Demographics of each study were extracted to include information on patient age, sex, and pathology. Data regarding the translation and cross-cultural adaptations were extracted to assess each design. The translation methods for each study were classified according to the *Guidelines for the process of Cross-Cultural Adaption of Self-report Measures* [11]*.* These cross-cultural adaption guidelines state an accurate translation must include an initial translation, synthesis of translations, back-translations, reviews by the expert committee and the pre-test version of the instrument. We also extracted data relating to the measurement properties of each study. These measurement properties were evaluated according to the *Quality Criteria for Measurement Properties of Health Status Questionnaires* [10]. This quality criteria evaluates: construct validity, internal consistency, reproducibility (agreement and reliability), agreement, responsiveness and ceiling and floor effects. Other measurement properties such as content validity and interpretability are only relevant to the development of original questionnaires, and therefore, not relevant to the scope of this review. Additionally, item criterion validity is measured when there is a gold standard of criteria available for comparison [6]. Shoulder assessments do not have a gold standard criteria for item selection, therefore, this property was excluded from the review. Tables were used to describe both the quality of testing and clinimetric results. This approach has been frequently used in a variety of systematic reviews for health–related questionnaires [6–8]. See Additional file 1 for further information on scoring systems.

Data extraction and ratings were performed by the first author (R.F.) and then reviewed by an independent reviewer (G.N.). Any disagreements between the rater and independent reviewer were discussed to reach a consensus. Any disagreements in data extraction and ratings were discussed with the third and senior author (J.M.) to reach a consensus.

### Limitations

In this study, the limitations lie within the inclusion criteria, as this review was limited to the use of peer-reviewed journal articles only. Keeping consistent with other published systematic review protocols [6–8], this excluded original versions of dissertations and theses with unpublished data regarding measurement properties. While a grey literature search was done through Google scholar, no results were found applicable for this review.

## Results

From the search strategies, 113 studies were retrieved but only 14 met eligibility criteria. The 14 versions represent 11 different languages/cultures; Chinese [15], Dutch [16–18], French-Canadian [13], Japanese [19], Norwegian [20], Persian [21], Polish [22], Portuguese-Brazilian [23, 24], Spanish [25], Swedish [26] and Turkish [27]. There was more than one study reporting clinimetric testing of the Dutch [16–18] and Portuguese-Brazilian [23, 24]. All Dutch versions were conducted independently; Wiertsema et al. reported on the reproducibility and translations of the WORC [16], Wessel et al. reported on the reliability, reproducibility and cognitive interviewing of creating a conceptually equivalent version [17] and de Witte et al. reported on the reliability and responsiveness of the WORC [18]. The Portuguese-Brazilian versions were conducted by the same group of researchers, however, one study focused on only the cross-cultural adaption process [24] and the other study focused on the evaluation of the psychometric properties [23].

Table 1 shows the demographic characteristics of the respective populations tested in the 14 studies. All studies included both male and female participants. While the literature recommends a minimum sample size of 100 patients, there are some exceptions [28]. For example, when evaluating content validity with qualitative methods, a sample size under 100 is justified [28]. In this review, all studies except the Portuguese –Brazilian [23] study (*n* = 30) had more than 50 patients. Patients were treated for a partial or a full rotator cuff tear, tendinopathy, impingement syndrome or calcific tendonitis.

**Table 1:** Demographic and clinimetric characteristics of the study populations from each study

Study Country (Language)	Year	Sample size(n)	Mean (SD) age	%female	%male	Shoulder condition
China (Chinese) [15]	2017	152	47.3 (9.5)	44.4	55.6	RC disorders that needed arthroscopic surgery
Netherlands (Dutch) [16]	2013	52	54.2 (9.7)	58	42	Partial or full thickness RC rupture, calcific tendonitis, or RC tendinopathy
Netherlands (Dutch) [17]	2013	57	53	47	53	RC tear, calcific tendonitis, impingement/tendinosis/ tendonitis
Netherlands (Dutch) [18]	2012	92	55 (8.7)	53	47	RC tear, calcific tendonitis, impingement
Canada (French –Canadian) [13]	2015	87	49.7 (12.4)	34.5	65.6	Tendinopathy, full or partial thickness RC tear
Japan (Japanese) [19]	2013	75	63.4 (11.1)	43	57	Impingement syndrome, tendinopathy, partial or full thickness RC tear
Norway (Norwegian) 19	2008	74	51 (11)	64	36	Shoulder pain or full-thickness rotator cuff tear
Iran (Persian) [21]	2009	120	46.7 (15.4)	45.6	48.7	Rotator cuff tendonitis, rotator cuff tendinosis with no tear, partial tear or full-thickness tear
Poland (Polish) [22]	2018	69	55.5	29	71	Had to be operated for rotator cuff disorders
Brazil (Portuguese-Brazilian) [ 23]	2008	100	56.7 (10.8)	69	31	Tendinopathy, full or partial thickness RC tear
Brazil (Portuguese-Brazilian) [24]	2006	30	55.1 (10.8)	46.7	53.3	Tendinopathy, full or partial thickness RC tear
Spain (Spanish) [25]	2015	60	57 (12.3)	44	56	Tendinopathy, full or partial thickness RC tear
Sweden (Swedish) [26]	2016	65	60	42	58	Surgery for subacromial pain condition or RC disorder
Turkey (Turkish) [27]	2006	72	54.9 (9.9)	75	25	Impingement syndrome, full or partial RC tears

Table 2 describes the ratings of the cross-cultural adaptions according to the *Guidelines for the Process of Cross-Cultural Adaptions of Self-Report measures* [11]. From the 14 eligible studies, 10 studies performed 100% of all the recommended cross-cultural adaption guidelines when performing the initial step of translation [13, 15–17, 19–21, 24, 25, 27]. These 10 studies also performed 100% of all recommended cross-cultural adaption guidelines for the step of synthesis [13, 15–17, 19–21, 24, 25, 27]. All of the back-translation step was preformed according to the cross-cultural adaption guidelines by 9 studies [13, 15–17, 19–21, 25, 27]. The Portuguese-Brazilian [24] study performed 50% of the back-translation step according to cross-cultural adaption guidelines, as they did not have two translators in the process. The step of the expert committee review was performed by 10 studies at 100%, according to the cross-cultural adaption guidelines [13, 15–17, 19–21, 24, 25, 27]. Furthermore, 9 studies performed 100% of the cross-cultural adaption guidelines for the step of pre-testing [13, 15–17, 20, 21, 24, 25, 27]. The Japanese [19] study performed 50% of the cross-cultural adaption guidelines for the step of pre-testing, as they did not provide the sample size used for pilot testing their questionnaire. The Dutch [18], Polish [22], Portuguese-Brazilian [23] and Swedish [26] studies used pre-translated versions of their questionnaires and therefore, did not report the translation process. Translation guidelines proposed by Guillemin, Bombardier and Beaton [11] were used by 13 out of 14 studies [13, 15–26] . While, the Turkish [27] study referred to the guidelines by Acquadro C, Jambon B, Ellis D, and Marquis P [29].

**Table 2:** Cross- cultural adaptions of the WORC into different languages that used the translation-based approach related to the Guidelines for the Process of Cross-Cultural Adaption of Self-Report Measures

Studies	Translation	Synthesis	Back translation	Expert committee review	Pretesting
China [15]	+	+	+	+	+
Dutch [16]	+	+	+	+	?
Dutch [17]	+	+	+	+	+
Dutch [18]	N/A	N/A	N/A	N/A	N/A
French –Canadian [13]	+	+	+	+	+
Japanese [19]	+	+	+	+	?
Norwegian [20]	+	+	+	+	+
Persian [21]	+	+	+	+	+
Polish [22]	N/A	N/A	N/A	N/A	N/A
Portuguese-Brazilian [ 23]	N/A	N/A	N/A	N/A	N/A
Portuguese-Brazilian [24]	+	+	?	+	+
Spanish [25]	+	+	+	+	+
Swedish [26]	N/A	N/A	N/A	N/A	N/A
Turkish [27]	+	+	+	+	+

*N/A* not applicable – The cross-cultural adaptions was not performed, only the clinimetric tests. Questionnaires used in these studies have been previously translated. + Positive rating, = negative rating; 0 no information available;? unclear

Table 3 presents the ratings of the evaluated measurement properties according to the *Quality Criteria for Measurement Properties of Health Status Questionnaire* [10] for each study. Overall, 13 studies evaluated the measurement property of reliability [13, 15–21, 24–27]. These 13 studies followed 100% of the quality criteria for measuring reliability; using test re-test and Cronbach’s alpha respectively. The measurement property of agreement was not adequately evaluated in any of the studies. Furthermore, 62% of studies [13, 15–17, 19, 20, 26, 27] followed 50% of the quality criteria, as they had designs where the minimal important change (MIC) was not defined and there were no convincing arguments that stated agreement to be acceptable. These studies reported agreement through standard error of the mean (SEM) or minimal detectable change (MDC) values, instead of MIC values. Additionally, 43% of studies [18, 21–23, 25] did not provide any information or evaluate the measurement property of agreement in their study. Only the French-Canadian and Swedish studies [13, 25] followed 100% of the quality criteria when measuring the property of internal consistency. Out of 14 studies, only 11 [15–23, 26, 27] performed 50% of the steps according to the quality criteria, as they did not include a factor analysis. Only the French-Canadian study [13] was able to follow 100% of the quality criteria when evaluating the measurement property of responsiveness. Only 50% of the recommended quality criteria was completed by 5 studies [15, 19, 20, 22, 26] when evaluating the property of responsiveness. These studies had designs in which the smallest detectable change group was bigger than the MIC OR the MIC and/or limits of agreement (LOA) were less than 1.96. Furthermore, 7 studies did not report the measurement property of responsiveness. All quality criteria steps for followed by 6 studies [13, 15, 19, 20, 22, 25] when evaluating construct validity. However, 7 studies did not evaluate or report the measurement property of construct validity [16–18, 21, 23, 26, 27]. The Chinese [15], Polish [22], Norwegian [20], Swedish [26], Dutch [17] and French-Canadian [13] studies followed 100% of the quality criteria for assessing the measurement property of ceiling or floor effects. The Persian study [21] followed 50% of the quality criteria when measuring ceiling and floor effects, as more than 15% of the respondents achieved the highest or lowest possible scores, despite having an adequate design and method. Furthermore, 54% of studies did not report any floor or ceiling effects [16, 19, 23–25, 27].

**Table 3:** Measurements properties of the WORC adapted into different languages related to Quality Criteria for Measurement Properties of Health Status Questionnaires

Studies	Reproducibility (Agreement)	Reproducibility (Reliability)	Internal Consistency	Responsiveness	Construct Validity	Ceiling and floor effects
China [15]	?	+	?	?	+	+
Dutch [16]	?	+	?	0	0	0
Dutch [17]	?	+	?	0	0	+
Dutch [18]	0	+	?	+	?	+
French –Canadian [13]	?	+	+	+	+	+
Japanese [19]	?	+	?	?	+	0
Norwegian [20]	?	+	?	?	+	+
Persian [21]	0	+	?	0	0	–
Polish [22]	0	+	?	+	+	+
Portuguese-Brazilian [ 23]	0	+	?	0	0	0
Portuguese-Brazilian [24]	0	0	0	0	0	0
Spanish [25]	0	+	+	0	+	0
Swedish [26]	?	+	?	–	0	+
Turkish [27]	?	+	?	0	0	0

*N/A* not applicable – The cross-cultural adaptions was not performed, only the clinimetric tests. Questionnaires used in these studies have been previously translated. + Positive rating, − negative rating, 0 no information available;? unclear

## Discussion

This systematic review evaluated the cross-cultural adaption procedures and measurement properties reported in 14 adapted versions of the WORC [13, 15–27]. As demonstrated in this review, the WORC is the superior choice of PRO for evaluating rotator cuff pathology, regardless of culture. However, the findings demonstrate that regardless of adaption methods used, there was a lack of clinimetric testing in the majority of translated versions of the WORC. Therefore, further validation of these adapted measures is needed to ensure they are able to measure the intended construct.

The primary outcome of the WORC is to evaluate disability related to RCDs and its effects on health-related quality of life [5]. Therefore, the intended patient population includes acute rotator cuff tendinitis, rotator cuff tendinosis with no tear, partial and full thickness tears and rotator cuff tear arthropathy [5]. While the majority of studies in this review recruited from this spectrum, some studies included calcific tendonitis [16–18]. It is important to highlight that calcific tendonitis does not fall under the scope of rotator cuff pathology, as it occurs from cell-mediated calcification inside the tendon. This can lead to patients experiencing extreme symptoms of pain and impingement, therefore, being confused with rotator cuff tear or impingement syndrome [30]*.* While the co-existence of calcific tendonitis with rotator cuff tear is not uncommon, calcific tendonitis is a non-degenerative condition that does not result in the tendon becoming torn or pathologic [30, 31]. Since the WORC is specific to rotator cuff pathology, inclusion of these patients hinder the homogeneity of the sample. Therefore, researchers should always recruit study populations that preserve the intended meaning of the outcome measure [32].

One issue that made the ratings less certain, was the lack of detail provided for the cross-cultural adaption processes used in the individual studies. Five studies [17, 19, 21, 24, 26] in this review provided a brief explanation of the translation processes. The Dutch [17] and Portuguese-Brazilian studies [24] assessed content validity by using cognitive interviewing. The results from the interviews demonstrated that the adapted WORC was only a reliable measure for patients, once cultural modifications had been applied to the individual items. Therefore, it is highly recommended to provide all relevant details of the translation process and discuss all issues that may have occurred, so that future researchers can anticipate when translating. In order to ensure that items fit the context of the culture, many researchers will change individual words or sentence structure. For example, the Chinese study [15] noted issues with translations of item 17. As most families in China are traditional, the term “rough-housing or horsing around” is inapplicable and had to be modified to the Chinese culture. Therefore, while researchers modify items that do not fit the context or culture of the target population, it must be done carefully to ensure that content validity is retained.

The back-translation step is often overlooked, but is critical according to the International Society for Pharmacoeconomics and Outcomes Research (ISPOR) ‘s guidelines [33]. Currently there is little agreement on how the back translation should be performed, but one of the translators should be of the origin language. This is to limit the amount of words or phrases that may not respect the speech patterns or colloquialisms of the target culture. For example, since there are a variety of dialects in Portuguese, the Portuguese-Brazilian version would have to be translated again to be used in Europe. ISPOR guidelines recommend that health-related PROs use conceptual translations, as they deal with subjective terms [33]. Therefore, researchers should adapt accordingly to maintain the intended meaning of the construct [16, 17, 34].

Reliability was evaluated in all studies and performed correctly according to the quality criteria. All studies in this review reported an Interclass Correlation Coefficient (ICC) value of over 0.70, which the quality criteria rates as excellent [11]. However, only the French–Canadian [13], Japanese [19] and Dutch [16] studies provided the type of ICC model and/or give a description of the confidence interval used. Reporting the type of ICCs used is important to distinguish results that maybe under - or overestimated. According to the quality criteria, reliability established by McGraw and Wong is preferred as systematic differences are considered to be part of the measurement error [11, 35]. The quality criteria also defines reliability by having an adequate measurement interval [11]. Therefore, a time period between the repeated administrations should be long enough to prevent recall, but short enough to ensure that clinical change has not occurred. Generally, 1 to 2 weeks is appropriate, but there could be reasons to choose otherwise [11]. Some studies [13, 21, 23] in this review had a time interval that was too long or not long enough. However, they were able to justify that due to participants starting rehabilitation immediately after their initial evaluation, researchers needed to either extend or shorten the time intervals to maintain consistency. Therefore, it is important for studies to describe and justify their time period to ensure that patients have not been changed on the construct that is being measured [36].

Agreement is another important measurement property that further evaluates the degree of which repeated measures applied to patients provide similar answers. It is easier to clinically interpret than the property of reliability, and provides the absolute error of measurement [11]. In this review, no study was able to fully evaluate agreement according to the quality criteria. The quality criteria recommend that studies should determine the MIC value because distribution-based methods do not provide a good indication of the importance of the observed change; however, studies in this review only report MDC values [6, 11]. Ideally, studies should test reproducibility by assessing both reliability (relative error of measure) and agreement (absolute error of measure) [6].

According to the quality criteria, responsiveness is a measure of longitudinal validity, and should be able to distinguish clinically important change from measurement error [11]. Responsiveness was assessed by 7 studies [13, 15, 18–20, 22, 26] and only the French- Canadian [13] and Dutch [18] studies reported responsiveness at 100% according to the quality criteria. These studies were able to report MIC values that were greater than the SDC, which were consistent with Kirkley et al. [5] However, it is important to note that there is more than one way to evaluate responsiveness according to the quality criteria. The area under the receiver operating characteristics curve (AUC), which measures the ability to distinguish patients who have and have not changed according to an external criterion, is also acceptable. An AUC value of at least 0.70 is considered to be adequate [11]. Therefore, researchers should always try to find a way to report the responsiveness in order to certify that the translated measures can detect patient improvement.

Ceiling and floor effects are another important measurement property according to the quality criteria [11]. Ceiling or floor effects are present if more than 15% of patients achieve the lowest or highest possible score, respectively. In this review, only 7 studies [13, 15, 17, 20–22, 26] reported testing for ceiling and floor effects. If ceiling or floor effects were present, content validity, reliability and responsiveness are all negatively impacted [6–8]. This indicates that the highest and lowest scores cannot be distinguished from each other, and changes cannot be measured in these patients. Therefore, reporting floor or ceiling effects verifies if the translated measures would fail to detect patient improvement or deterioration [6].

Construct validity was performed according to quality criteria in only a few studies [13, 15, 19, 20, 25]. These studies formulated hypotheses concerning the concepts measured. The most important feature of construct validity is to formulate hypotheses α priori, and to specify the direction of the expected correlation and its magnitude. Stating the hypothesis is crucial, otherwise the risk of bias is high, and it would be easier to develop an alternative explanation for the low correlations, than to admit that the construct validity has been compromised [6, 11].

This review demonstrates that there were many inconsistencies with some of the reported measurement properties in the various adaptions of the WORC. In the systematic review of the cross-cultural adaption and measurement properties of the McGill Pain Questionnaire [8], it was observed that many properties were either not evaluated or inappropriately measured. This was also similar to findings of a systematic review that looked at cross-cultural adaptions and measurement properties of various shoulders outcomes in Portuguese [6]. The lack of appropriately testing these measures creates challenges for researchers and clinicians. The goal with adapting validated PROs is to achieve equivalence. Therefore, researchers must focus on maximizing both the linguistic, cultural and structural system of health-related measurements [6]. By developing culturally equivalent versions of these instruments, we can promote the exchange of information from studies across different cultures, without constantly having to create new PROs [6–8]. Therefore, following the proper guidelines for cross-cultural adaptations and for testing measurement properties is critical.

Based on the findings from this review, the French-Canadian study [13] had performed the most successful according the quality criteria and the cross-cultural adaption guidelines. However, just because a study received the highest number of positive ratings, does not necessarily mean it is the best outcome measure. Ratings depend on the availability of information and the quality of reporting on the assessment. For example, newer outcome measures may have many indeterminate ratings of measurement properties, as they are yet to be evaluated. Furthermore, it is important to note that there is no overall quality score with these guidelines [10, 11], as often done in systematic reviews of randomized clinical trials. Having an overall quality score assumes that all measurement properties are equally important, which is not always true. A successful outcome measure requires a variety of different qualities with respect to reproducibility and responsiveness [11]. In particular, evaluative PROs such as the WORC, require a high level of agreement to be able to measure important changes, which was lacking in the present studies [11].

Finally, this review demonstrated that while the WORC is a favourable tool for measuring QoL for rotator cuff disorders, there are other disease specific instruments such as the Rotator Cuff Quality of Life Index (RC-QOL). However, these two instruments differ by the items they are trying to evaluate. The RC-QOL focuses on more physically demanding activities such as mopping the floor, carrying 10lbs etc. unlike the WORC [37]. Furthermore, the scoring for both outcome measures differ as the RC-QOL rates from 0 to 100, with a lower score meaning a lower quality of life, which is the inverse for the WORC [5, 37]. Similar to the WORC, the RC-QOL is also adapted for different cultures [37, 38]. Therefore, future studies should further investigate the differences and similarities of both adapted measures, to fully evaluate if the intended constructs are being retained.

## Conclusion

The WORC was able to be successfully translated for different cultures, however, the evaluation of the measurement properties was not sufficient. Therefore, further validation of the adapted versions of the WORC is required before routine use in clinical practice. This review has shown that by continuing to adapt the WORC, more cultures will be able to benefit from this PRO.

## Supplementary information

**Additional file 1: Figure S1.** Scoring system for the cross-cultural adaptions.

## Data Availability

All data generated or analyzed during this study are included in this published article.

## Abbreviations

AUC: Area under the receiver operating characteristics curve
ICC: Interclass correlation coefficient
ISPOR: International society for pharmacoeconomics and outcomes research
LOA: Limits of agreement
MDC: Minimal detectable change
MIC: minimal important change
PRO: Patient reported outcome
QOL: Quality of life
RCD: Rotator cuff disorder
RC-QOL: Rotator cuff quality of life index
SEM: Standard error of the mean
WORC: Western Ontario Rotator cuff index
