# Supplementary material for: Cross-cultural adaptions and measurement properties of the WORC (Western Ontario rotator cuff index): a systematic review
Source: Health Qual Life Outcomes. 2020 Jan 29;18:17. doi: 10.1186/s12955-020-1276-9 (PMC6988228; doi:10.1186/s12955-020-1276-9)
Supplement: Supplementary file 1 — Additional file 1: Figure S1. Scoring system for the cross-cultural adaptions. [file 12955_2020_1276_MOESM1_ESM.docx]

**TABLE 1**. Guidelines for the process of the cross-cultural adaption of self-reported measures^11^(adapted from Costa et al.^8^).

| Steps | Description | Rating Scheme |
| --- | --- | --- |
| Translation | Two (or more) translators should independently translate the original questionnaire. The translators should preferably be native speakers to target language. | + Translation performed by at least two independent translators  ? Doubtful translation procedure  - Translation performed by only one translator  0 No information about translation |
| Synthesis | The translators should synthesize the multiple translations to produce a consensus of the translations. | + Performed synthesis  ? Doubtful design  0 No information about synthesis OR translation performed by only one translator. |
| Back Translation | Translators, blinded to the original questionnaire shoulder translate the consensus translation back into the original language. | + Back translation performed by at least two independent translators  ? Doubtful back translation procedure  - Back translation performed by only one translator  0 No information about back translation |
| Expert Committee | The expert committee should consolidate all the versions of the questionnaire and develop what would be considered the prefinal version of the questionnaire for testing. | + Clearly reported the existence of an expert committee  ? Doubtful design  0 No information about the expert committee |
| Pretesting | The prefinal questionnaire undergoes pilot testing with members of the target population. | + Performed pretesting  ? Doubtful design  0 No information |

+= positive rating; - = negative rating; 0= no information available; ?=unclear

**TABLE 2**. Quality Criteria for measurement properties of health status questionnaires^10^ (adapted from Costa et al.^8^).

| Property | Definition | Quality Criteria |
| --- | --- | --- |
| Internal Consistency | Internal consistency is a measure of the homogeneity of a (sub) scale. It indicates the extent to which items in a (sub)scale are intercorrelated, thus measuring the same construct. Factor analysis should be applied to determine the dimensionality of the item-this is, to determine whether or not they formed only one overall dimension or more than one. | + Factor analyses performed on adequate sample size (7 x # items and > 100) AND Cronbach’s alpha(s) calculated per dimension AND Cronbach’s alpha(s) between 0.70 and 0.95;  ? No factor analysis OR doubtful design or method;  - Cronbach’s alpha(s) <0.70 or .0.95, despite adequate design and method;  0 No information found on internal consistency |
| Construct Validity | Content validity examines the extent to which scores on a particular questionnaire relate to other measures in a manner that is consistent with theoretically derived hypotheses concerning the concepts that are being measured. | + Specific hypotheses were formulated AND at least 75% of results are in accordance with these hypotheses;  ? Doubtful design or method (e.g, no hypotheses);  - Less than 75% of hypotheses were confirmed, despite adequate design and methods;  0 No information found on construct validity |
| Reproducibility | The degree to which measurements in stable persons (test- retest) provide similar answers. |  |
| Reliability | The extent to which patients can be distinguished from each other, despite measurement errors. (relative measurements error) | + ICC or Kappa > 0.70;  ? Doubtful design or method (e.g., time interval not mentioned);  - ICC or Kappa <0.70, despite adequate design and method;  0 No information found on reliability |
| Agreement | The extent to which the scores on repeated measures are close to each other. | + MIC <SDC or MIC outside the LOA or convincing arguments that agreement is acceptable;  ? Doubtful design or method or (MIC not defined AND no convincing arguments that agreement is acceptable);  - MIC > SDC or MIC equals or inside LOA, despite adequate design and method;  0 No information found on construct validity |
| Responsiveness | The ability of a questionnaire to detect clinically important change over time in the concept being measured. A predefine hypothesis about the relation of change in the instrument to corresponding changes in the reference measures should be postulated. | + Smallest detectable change individual or Smallest detectable change < Minimal import change OR Minimal important change outside the limits of agreement OR Responsiveness ratio  > 1.96 OR Area under the curve > 0.70;  ? Doubtful design or method OR sample size < 50 or methodological flaws;  - Smallest detectable change individual or Smallest detectable change group > Minimal important change OR Minimal important change equals or inside limits of agreement or Responsiveness ratio < 1.96 OR Area under the curve <0.70 despite adequate design and methods;  0 No information found on responsiveness. |
| Floor and Ceiling Effects | The number of respondents who achieved the lowest or highest possible score. | + < 15% of the respondents achieved the highest or lowest possible scores;  ? Doubtful design or method OR sample size <50 OR methodological flaws;  - > 15% of the respondents achieved the highest or lowest possible scores, despite adequate design and methods;  0 No information found on interpretation. |

+ = positive rating; ?=doubtful design or method; - =negative rating; 0 = no information available. Doubtful design or method = lacking of a clear description of the design or methods of the study, smaller than 50 subjects or any important methodological weakness in the design or execution of the study. MIC =minimum important changes; SDC = smallest detectable change; LOA= limits of agreement; ICC = interclass correlation coefficient; SD = standard deviation.
